# Microbial communities in the nepheloid layers and hypoxic zones of the Canary Current upwelling system

**DOI:** 10.1002/mbo3.705

**Published:** 2018-10-11

**Authors:** Stefan Thiele, Andreas Basse, Jamie W. Becker, Andre Lipski, Morten H. Iversen, Gesine Mollenhauer

**Affiliations:** ^1^ Max‐Planck‐Institute for Marine Microbiology Bremen Germany; ^2^ Alfred Wegener Institute for Polar and Marine Research Bremerhaven Germany; ^3^ Department of Biology Haverford College Haverford Pennsylvania; ^4^ Department of Food Microbiology and Hygiene Rheinische Friedrich‐Wilhelms Universität Bonn Bonn Germany; ^5^ MARUM and University of Bremen Bremen Germany; ^6^ Friedrich Schiller University Jena Germany

**Keywords:** bacterial community, CARD‐FISH, fatty acid methyl ester, hypoxic layers, nepheloid layer, SAR11 clade

## Abstract

Eastern boundary upwelling systems (EBUSs) are among the most productive marine environments in the world. The Canary Current upwelling system off the coast of Mauritania and Morocco is the second most productive of the four EBUS, where nutrient‐rich waters fuel perennial phytoplankton blooms, evident by high chlorophyll a concentrations off Cape Blanc, Mauritania. High primary production leads to eutrophic waters in the surface layers, whereas sinking phytoplankton debris and horizontally dispersed particles form nepheloid layers (NLs) and hypoxic waters at depth. We used Catalyzed Reporter Deposition Fluorescence In Situ Hybridization (CARD‐FISH) in combination with fatty acid (measured as methyl ester; FAME) profiles to investigate the bacterial and archaeal community composition along transects from neritic to pelagic waters within the “giant Cape Blanc filament” in two consecutive years (2010 and 2011), and to evaluate the usage of FAME data for microbial community studies. We also report the first fatty acid profile of *Pelagibacterales strain* HTCC7211 which was used as a reference profile for the SAR11 clade. Unexpectedly, the reference profile contained low concentrations of long chain fatty acids 18:1 *cis*11, 18:1 *cis*11 11methyl, and 19:0 cyclo11–12 fatty acids, the main compounds in other *Alphaproteobacteria*. Members of the free‐living SAR11 clade were found at increased relative abundance in the hypoxic waters in both years. In contrast, the depth profiles of *Gammaproteobacteria* (including *Alteromonas and Pseudoalteromonas*), *Bacteroidetes*,* Roseobacter*, and *Synechococcus* showed high abundances of these groups in layers where particle abundance was high, suggesting that particle attachment or association is an important mechanisms of dispersal for these groups. Collectively, our results highlight the influence of NLs, horizontal particle transport, and low oxygen on the structure and dispersal of microbial communities in upwelling systems.

## 1 INTRODUCTION

Eastern boundary upwelling systems (EBUS) can be found off the coast of Peru, the USA, Namibia, and Morocco/Mauretania. EBUS are among the most productive marine environments in the world, accounting for ~10% of global ocean primary production (Behrenfeld & Falkowski, 1997). Due to the persistent upwelling of nutrient‐rich waters, these upwelling systems harbor perennial phytoplankton blooms (Carr & Kearns, 2003; Carr et al., 2006; Gattuso, Frankignoulle, & Wollast, 1998). The high productivity results in extensive vertical and horizontal carbon transport (Arístegui et al., 2004), with importance for global carbon cycling. The Canary Current upwelling system (CC) off the coast of Morocco and Mauretania is the second most productive of the EBUS (Carr, 2001; Lachkar & Gruber, 2011). In the southern part of the CC off Cape Blanc (Mauretania) coastal upwelling of cold water sustains a year round phytoplankton bloom with highest productivity from January through June (Arístegui et al., 2009; Lathuilière, Echevin, & Lévy, 2008). Due to the off‐shore Ekmann transport the coastal phytoplankton biomass production forms “giant Cape Blanc filament” that extend several hundred kilometers offshore, the largest filament in all EBUS (Van Camp, Nykjaer, Mittelstaedt, & Schlittenhardt, 1991). The high primary production results in large vertical export of organic matter via settling of marine snow and fecal pellets thereby forming the “biological carbon pump”.

Horizontal Ekmann transport from the coast offshore combined with low particle sinking rates of ~5 m d^−1^ causes the formation of an intermediate nepheloid layer (INL) between ~200 m and ~650 m depth (Karakaş et al., 2006). Additionally, a bottom layer (BL) 50–100 m above the seafloor is formed by resuspension from the slope and particles settling with 35 m d^−1^ (Fischer & Karakaş, 2009; Karakaş et al., 2006; Müller & Fischer, 2001). Often oxygen minimum zones (OMZs) or hypoxic waters are found within the INL between ~400 and 800 m depth (Fischer, Reuter, Karakas, Nowald, & Wefer, 2009).

In these waters bacteria are typically the main degraders of organic matter, causing a further depletion of oxygen in the surrounding waters within the INLs (Iversen, Nowald, Ploug, Jackson, & Fischer, 2010). This leads to changes in nutrient fluxes, mainly loss of nitrogen and the production of methane, nitrous oxide, and carbon dioxide (Wright, Konwar, & Hallam, 2012). Microbial diversity declines within OMZs (Beman & Carolan, 2013; Bryant, Stewart, Eppley, & DeLong, 2012).While *Cyanobacteria*,* Bacteroidetes*,* Rhodobacterales* (*Alphaproteobacteria*), and *Alteromonadales*, as well as SAR86 clade members (both *Gammaproteobacteria*) dominate in surface waters, they are less abundant in oxygen deprived waters, whereas members of the SAR11 clade of *Alphaproteobacteria* were found in abundance throughout the water column and also in hypoxic waters (Lüke, Speth, Kox, Villanueva, & Jetten, 2016; Ulloa, Canfield, DeLong, Letelier, & Stewart, 2012).

Enhanced surface primary production also enhances secondary production by the bacterial and archaeal community in surface waters (Alonso‐Sáez, Sánchez, & Gasol, 2012; Vaqué et al., 2014). When aggregates are formed and sink through the water column increased productivity may occur in deeper water layers (Baltar, Arístegui, Gasol, & Herndl, 2012). As zooplankton mainly feed in the surface layers, microbial activity becomes the dominant attenuation process of organic matter export at depth (Iversen et al., 2010; Stemmann, Jackson, & Gorsky, 2004). Previous studies have observed increased contribution from *Bacteroidetes*,* Gammaproteobacteria*, and *Rhodobacteraceae* (including the *Roseobacter* clade) to the microbial communities within the highly productive upwelling regions and during coastal phytoplankton blooms (Alonso‐Sáez et al., 2007; Teeling et al., 2012, 2016), pointing toward a connection between these groups and elevated primary productivity. In contrast, the open and oligotrophic Atlantic Ocean is often dominated by members of the SAR11 clade, whereas the abundance of *Bacteroidetes* is reduced (Alonso‐Sáez et al., 2012; Schattenhofer et al., 2009; Thiele, Fuchs, Ramaiah, & Amann, 2012). However, most studies have so far focused on bacterial and archaeal diversity in surface waters, whereas microbial studies in deeper layers has focused on specific groups and found high abundances of SAR202, *Crenarchaeota* Group I, or *Euryarchaeota* Group II (Varela, Van Aken, & Herndl, 2008; Varela, Van Aken, Sintes, & Herndl, 2008).

Here we report an investigation of the bacterial and archaeal community composition of depth profiles within two transects of the “giant Cape Blanc filament” conducted in two consecutive years. We used CARD‐FISH to analyze samples derived from depth profiles taken at six stations, and compared the results to profiles of fatty acid concentrations from the water column aimed to elucidate the bacterial community structure in the Canary Current upwelling system. In addition, we combined CARD‐FISH‐based community composition analyses with FAME‐based taxonomy to combine benefits from both methods for the characterization of marine microbial communities. Even though the SAR11 clade is the most abundant bacterial group in marine systems, the fatty acid composition of members of this clade remains unknown. Therefore, we analyzed the fatty acid profile of *Pelagibacterales strain* HTCC7211, a representative of the globally abundant Ia.3 subgroup of the SAR11 clade (Stingl, Tripp, & Giovannoni, 2007), to enhance FAME analyses for *Alphaproteobacteria* in marine environments.

## 2 MATERIALS AND METHODS

### 2.1 Sampling stations

**Figure 1:**
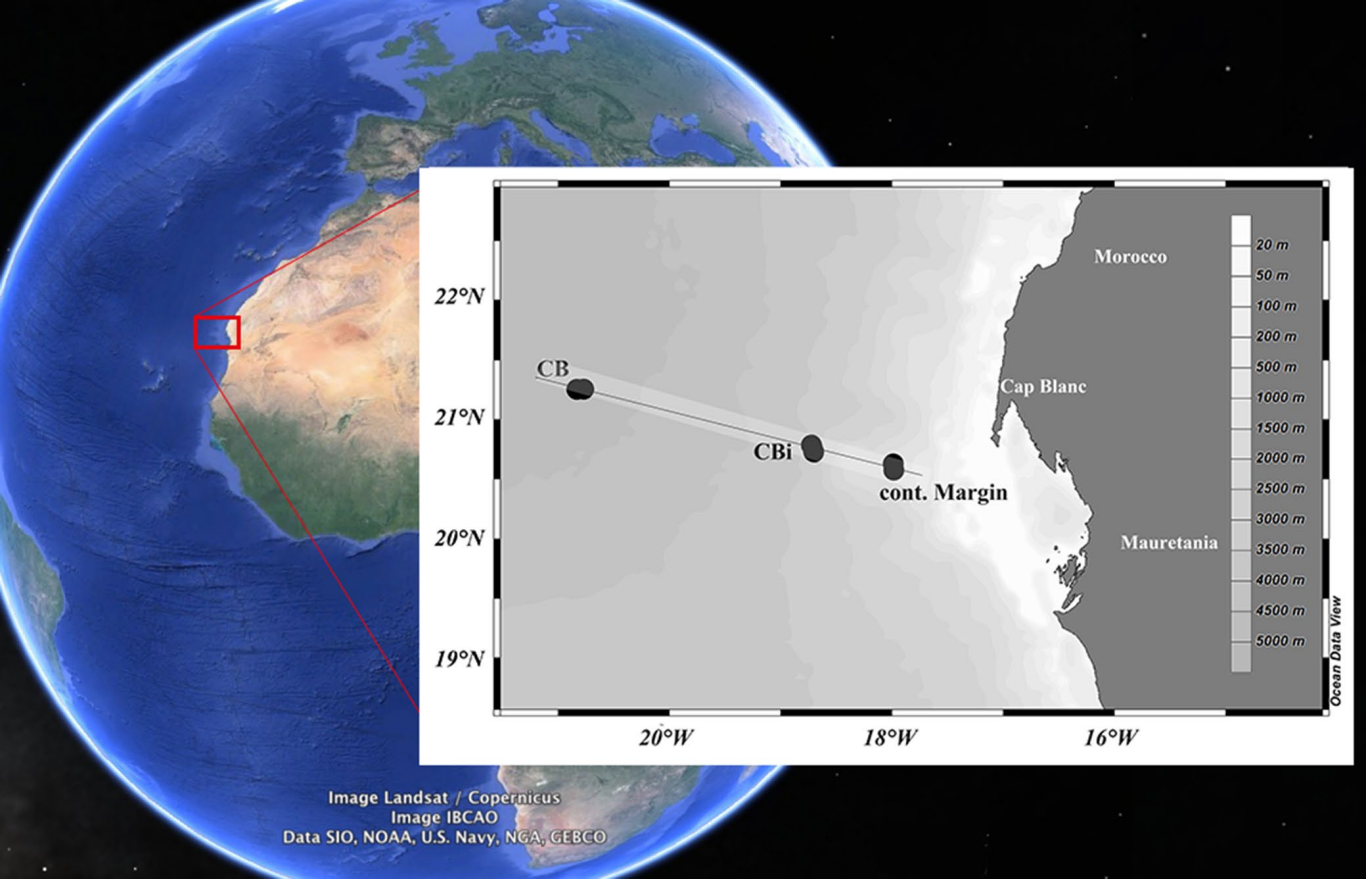
Map of the sampling area with the stations CB, CBi, and Continental Margin

Samples used in this study were taken on an east west transect in the CC region off Cape Blanc during two cruises on RV Poseidon and RV Maria S. Merian POS 396 from 24/02/2010 to 08/03/2010 and MSM 18‐1 from 17/04/2011 to 05/05/2017, as reported previously (Basse et al., 2014). Two well‐described mooring stations CB (outer mooring) and CBi (inner mooring) (Fischer et al., 2009) and one additional station were sampled during both cruises: GeoB14201 (station CB; open ocean; bottom depth 4,154 m), GeoB14202 (station CBi; bottom depth 2,700 m), and GeoB14207 (continental margin; bottom depth 764 m) during POS 396, and GeoB15709 (station CB; open ocean; bottom depth 4,156 m), GeoB15703 (station CBi; bottom depth 2,768 m), and GeoB15704 (continental margin; bottom depth 778 m) during MSM 18‐1 (Figure 1). Depth profiles of suspended particulate matter (SPM) and fatty acids were collected by in situ filtration using battery‐powered pumps (WTS 6‐1‐142LV; McLane Research Laboratories, Falmouth, MA) or from the vessel's underway water‐intake system. Sampling volumes were determined by the pump control software and via a mechanical flow meter. Samples for fatty acid analyses were collected on precombusted Whatman GF/F glass fiber filters with a diameter of 142 mm and a pore size of 0.7 μm, as previously described (Basse et al., 2014). In addition, for CARD‐FISH analyses seawater was sampled at different depths from the surface to the bottom layer using a CTD rosette sampler from which 100–200 ml water samples were fixed with 1% formaldehyde final concentration. The fixed samples were serially filtered with 10 and 3 μm polycarbonate membranes (Millipore, Billerica, USA). The final filtration step was done in duplicate with 20 ml to 100 ml of the prefiltered seawater on 0.22 μm pore size polycarbonate membranes (Millipore, Billerica, USA). All samples were stored at −20°C until processing.

### 2.2 Biogeochemical parameters

Water temperature, salinity, oxygen, turbidity, and chlorophyll fluorescence were measured with a self‐contained SBE‐19 CTD profiler equipped with a conductivity‐temperature‐depth probe plus oxygen sensor, a CHELSEA‐fluorometer, and a WETLABS turbidity sensor. The sensors were calibrated before the cruise, but the oxygen sensor could not be calibrated continuously during the POS396 cruise and consequently the measured values have to be interpreted as relative values.

### 2.3 Lipid extraction and analyses

Filters were stored at −20°C and dried immediately prior to extraction. Four pieces were cut out of the dried GF/F filters with a broach (∅ = 12 mm) for determining particulate organic matter content (POM), assuming that the composition of cut‐out filter pieces is representative of the entire filter. Lipids were extracted from the remaining filter parts as described previously (Basse et al., 2014). One subsample of a laboratory‐internal sediment standard was extracted every 11 samples using the same methods. Total lipid extracts (TLE) were saponified with 300 μl of 0.1 M KOH in MeOH with 10% H_2_O at 80°C for 2 hr. After that, ~80% of the solvent was evaporated using dried N_2_, and the neutral lipids were repeatedly extracted into hexane five times. The acid fraction containing the fatty acids was recovered five times in DCM after acidifying the solution to pH = 1 with hydrochloric acid. The fatty acids were methylated by adding 3.5 ml MeOH and 174 μl 37% HCl, replacing the air in the vial with N_2_ and reacting in the closed vial at 50°C over night. After cooling down solvents were evaporated down to a small rest volume of a few μl and re‐dissolved with ca. 200 μl DCM/Methanol (1:1). Serapure‐H_2_O and hexane were added and fatty acid methyl esters (FAMEs) were extracted into hexane for five times. Hexane was evaporated and FAMES were eluted with DCM/hexane 2:1 over self‐packed 6 mm diameter columns (4 cm of 1% H_2_O de‐activated SiO_2_, 0.063–0.2 mm mesh size, and 0.5 cm of Na_2_SO_4_.

For initial peak identification, an aliquot of some representative samples was silylated and analyzed on a gas chromatography mass spectrometer (GC‐MS; Agilent 7990B GC) coupled to Agilent 5977A MSD, Santa Clara, USA). To definitely distinguish between cyclopropyl FAs and unsaturated FAs with the same mass, selected samples were analyzed again after removing all unsaturated FAs on an AgNO_3_ column. After identification, FA‐concentrations were analyzed by capillary gas chromatography on a HP 5890 (POS396) and an Agilent 7890 (MSM18‐1) chromatograph each with a 60 m fused silica column (0.25 mm/0.25 μm), and flame ionization detection.

### 2.4 FAME analysis of *Pelagibacterales strain* HTCC7211

*Pelagibacterales strain* HTCC7211 (obtained from Stephen Giovannoni at Oregon State University) was grown in replicate 1 L batch cultures in AMS1 medium (Carini, Steindler, Beszteri, & Giovannoni, 2013) supplemented with 50 μM pyruvate, 50 μM glycine, and 10 μM methionine under constant illumination (2 μmol photons m^−2^ s^−1^) without shaking at 22°C for 9 days. Acid‐washed and autoclaved tissue culture‐grade polycarbonate was used for all cultures. Cell enumeration was determined using a Guava easyCyte 12HT benchtop flow cytometer (EMD Millipore) after staining with SYBR Green I nucleic acid stain (Lonza; 1% final concentration). Culture purity was monitored by flow cytometry and further tested using a suite of three broths: ProAC, ProMM, and MPTB (Berube et al., 2015; Morris, Kirkegaard, Szul, Johnson, & Zinser, 2008; Saito, Moffett, Chisholm, & Waterbury, 2002). Cells were harvested by centrifugation, washed in phosphate buffered saline and stored at −20°C. Fatty acid methyl esters were prepared from cell pellets as described by Sasser (1990). In brief, samples underwent saponification with 15% NaOH in 50% methanol and acid methylation with 6 N HCl in 50% methanol. The FAME extracts were analyzed using a Hewlett–Packard model 6890 gas chromatograph equipped with a 5% phenyl methyl silicone capillary column and a flame ionization detector. The identity of fatty acids was verified by GC‐MS with an Agilent model 7890A gas chromatograph equipped with a 5% phenyl methyl silicone capillary column and coupled with a model 5975C mass selective detector. The chromatographic conditions were used as described previously (Lipski & Altendorf, 1997). The positions of hydroxy, methyl, cyclopropane groups, and double bonds were determined from the carboxyl group of the fatty acid molecule according to the recommendations of the 1977 IUPAC‐IUB Commission on Biochemical Nomenclature (CBN).

### 2.5 CARD‐FISH

CARD‐FISH using probes specific for the investigated bacterial groups was done after a standard protocol according to Thiele (Thiele, Fuchs, & Amann, 2011) exactly as described in a previous study (Thiele, Fuchs, Amann, & Iversen, 2015). In brief, duplicate or triplicate samples from all depths were used to conduct CARD‐FISH with specific probes (Supporting Information Table S1) and subsequent DAPI staining. The counting was done using an automated counting routine based on the MPISYS software and subsequent image processing using the software ACMEtool2 after manual quality control (Bennke et al., 2016) and the relative abundance of the different probes was calculated based on DAPI counts. Posterior ANOVA tests were done using the software package R (R core team, 2014).

## 3 RESULTS

### 3.1 Oceanographic settings

**Figure 2:**
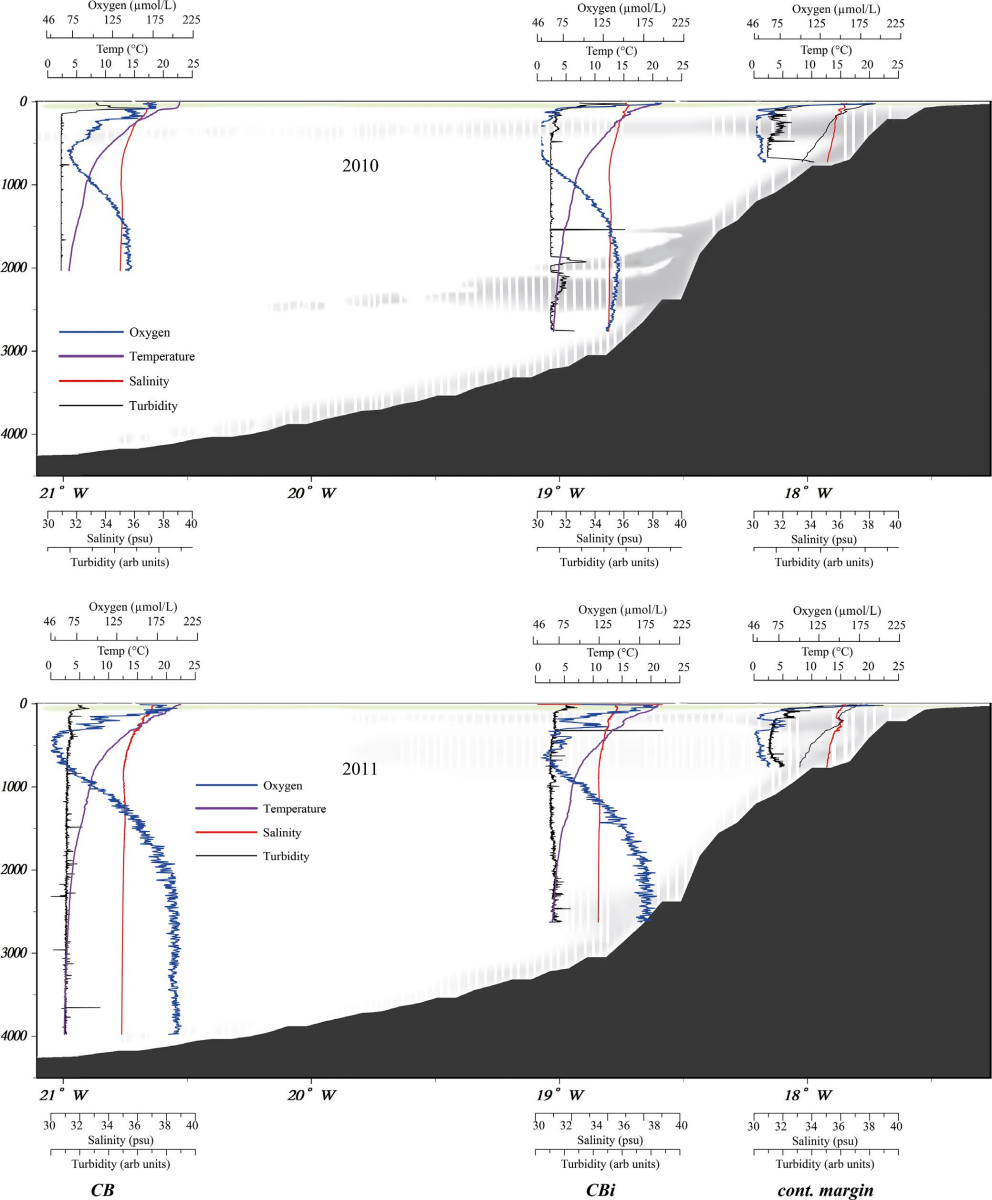
Depth profiles of Oxygen (blue), Temperature (purple), Salinity (red), and Turbidity (black) for 2010 (a) and 2011 (b) based on (Basse et al., 2014). The Chl a maximum is indicated as a green layer (data not shown) and nepheloid layers are indicated as gray layers

For this study, three factors were of importance, namely the chlorophyll a (Chl a) concentrations, turbidity, and oxygen concentrations. High Chl a concentrations indicate the existence of a phytoplankton bloom throughout the entire transect during both cruises with Chl a maxima between the surface and 85 m depth. Turbidity profiles in the filament off Cape Blanc indicated the presence of strong NLs, such as an INL between 250–600 m, BL clouds at 1,900–2,800 m, and a BL around 50–100 m above the seafloor (Figure 2). The lowest oxygen concentrations (~35.7–98.2 μM) were observed at depths of 300–600 m (Basse et al., 2014). Temperature profiles were similar between all stations, with ~22°C in the surface water, 8°C at 1,000 m depth, and 5°C in the deep ocean. Salinity decreased from ~36 at the surface to <35 below 500 m for all stations due to a change from South Atlantic Central Water to North Atlantic Central Water and North Atlantic Deep Water at depth (Iversen et al., 2010).

### 3.2 Bacterial and archaeal distribution

**Figure 3:**
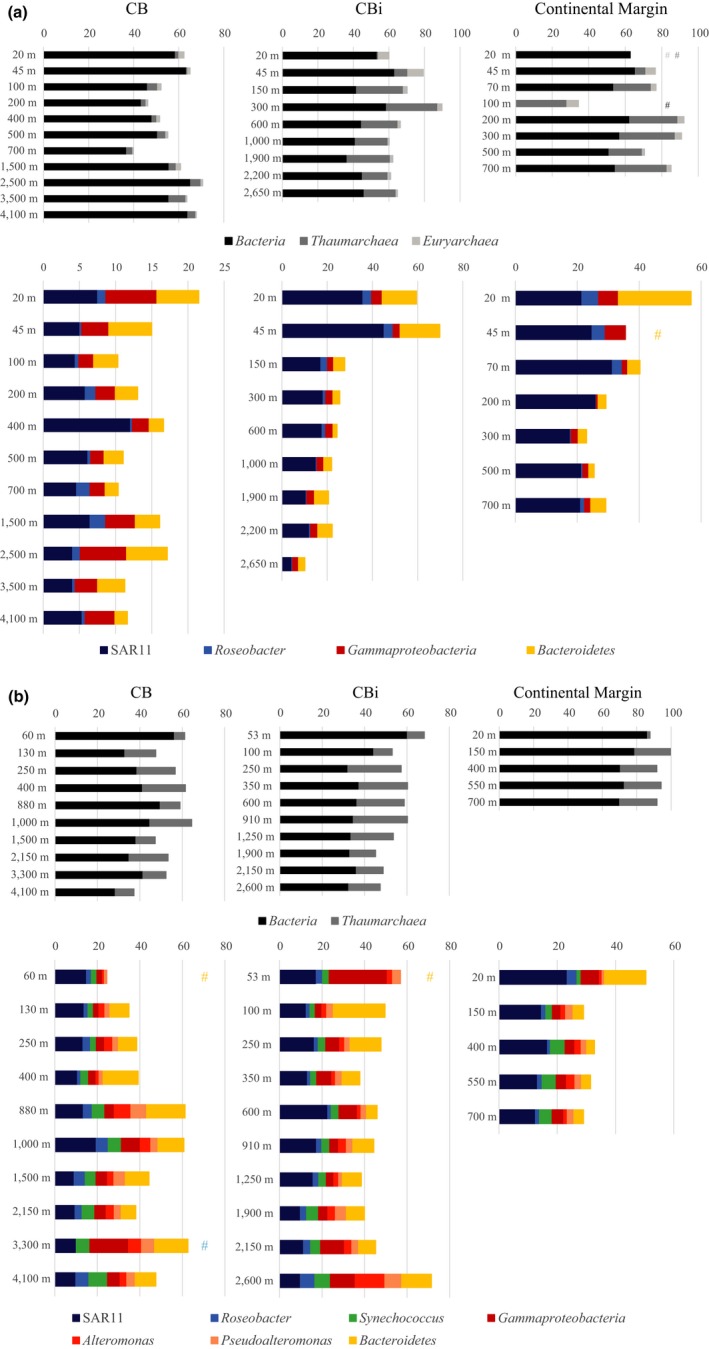
Depth profiles of the relative abundance of different bacterial and archaeal groups based on CARD‐FISH counts of the years 2010 (a) and 2011 (b). The hashes mark missing values, the color of the hash shows the missing bacterial group

We analyzed the bacterial and archaeal community composition using CARD‐FISH (Supporting Information Table S2) and fatty acid composition (*Bacteria* only; Supporting Information Table S3) for similar transects sampled in consecutive years. The total detection rate of *Bacteria*,* Thaumarchaea*, and Marine Group I *Euryarchaea* ranged between 40% and 90%. *Bacteria* and *Euryarchaea* (measured only in 2010) abundance were highest in surface waters decreased mostly after 100 m depth, whereas the abundance of *Thaumarchaea* increased below 100 m and often reaching 25% of relative abundance (Figure 3a and b). This general decrease in bacterial abundance with depth is also reflected in the decreasing amount of total and bacterial specific fatty acids with depth.

### 3.3 *Alphaproteobacteria*

The relative abundance of SAR11 bacteria was relatively low, particularly at station CB in 2010, where the relative abundance was only ~5%, with the exception of a peak of 12.0 ± 0.9% at 400 m depth (Figure 3a). In 2011 elevated relative abundances of 13.3 ± 4.6% and 19.5 ± 1.5% were found at 880 m and 1,000 m, at station CB (Figure 3b). A higher relative abundance of the SAR11 clade was found at station CBi at 600 m depth in 2011, with 22.6 ± 11.1% (Figure 3b). This pattern is not reflected in the depth profile of station CBi from 2010, where the overall highest values where found at the surface with 35.8 ± 7.4% and 45.1 ± 8.7% at 20 m and 45 m, respectively (Figure 3a). The relative abundance was significantly lower (ANOVA; *p* < 0.01) than at depths below 45 m, where it decreased from 17.1 ± 2.1% at 150 m to 4.3 ± 2.8% at 2,650 m (Figure 3a). At the continental margin, the relative abundance of SAR11 was relatively constant with ~23% in 2010, and at ~14% in 2011 (Figure 3a and b).

*Roseobacter*, the second group of *Alphaproteobacteria* investigated in the CC was found at a slightly higher relative abundance in 2010 than in 2011 and on the continental margin as compared to stations CB and CBi (Figure 3a and b). In 2010 the abundance at station CB ranged between 0.3% and 2.2%, whereas at stations CBi and the continental margin, the relative abundance was highest in the surface waters with ~4% and decreased to <1% at depth. In 2011, the relative abundance of *Roseobacter* decreased at all stations from the surface toward deeper waters. However, the highest relative abundances of *Roseobacter* were found to be 6.8 ± 3.5% at 2,600 m depth at station CBi and 6.2 ± 5.0% at 4,100 m at station CB (Figure 3b). In addition, another peak with ~5% was found between 880 and 1,500 m at station CB (Figure 3b).

**Figure 4:**
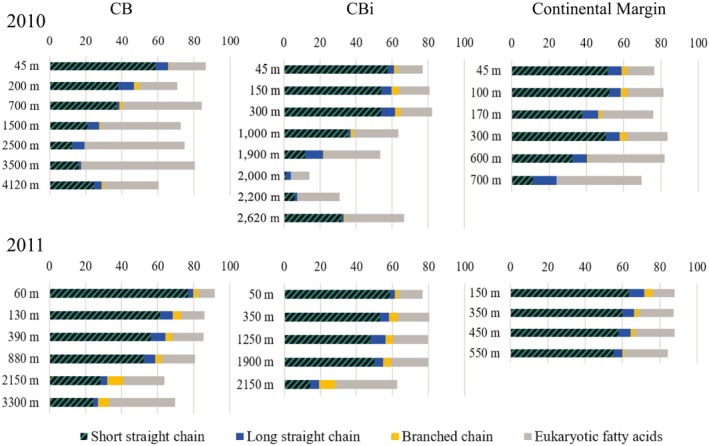
Depth profiles of the fatty acid abundance of all stations in the years 2010 and 2011. Dark blue and green stripes mark the short straight chain fatty acids (16:0, 16:1 cis9, 17:0 cyclo) mostly found in SAR11, *Synechococcus* and *Gammaproteobacteria*, blue mark the long straight chain fatty acids (18:1 cis11, 19:0 cyclo) mostly found in *Roseobacter*, orange mark the branched chain fatty acids (15:0 iso, 15:0 anteiso, 16:0 iso, 17:0 iso, 17:0 anteiso) mostly found in *Bacteroidetes*, and gray mark the fatty acids mostly found in eukaryotes (18:0, 18:1 cis9, 18:2 cis9,12)

The analysis of *Pelagibacterales strain* HTCC7211 lipid profile revealed a high percentage of the short chain fatty acids 16:0, 16:1 *cis*9, and 17:0 cyclo9‐10 (Table 1). In contrast, the fatty acid profiles of most other *Alphaproteobacteria* are dominated by longer chain fatty acids (hereafter referred to as “long chain”), namely 18:1 *cis*11 and its derivatives 18:1 *cis*11 11methyl and 19:0 cyclo11–12, which can constitute up to 90% of the total fatty acid content (Table 1 and references therein). While the lipid profile of *Oceanicaulis*, and *Pelagibaca* usually display between 20% and 80% of 18:1 *cis*11, *Roseobacter* have a particularly high content of 18:1 *cis*11 at 84%–90% (Table 1 and references therein). In all fatty acid profiles, the short straight chain fatty acids showed a higher abundance at the lower depths and declined in the higher depths. The abundance of long straight chain fatty acids did not show any correlation with depth but were present in all depths for all profiles (Figure 4).

**Table 1:** Fatty acid profiles of representative genera within the *Alphaproteobacteria*. Genus *Caulobacter* is type of the order *Caulobacterales* (*Alphaproteobacteria*). The genera *Roseobacter*,* Pelagibaca* and *Oceanicaulis* are members of *Rhodobacterales* and represent three genera comprising deep sea associated species. *n*: number of fatty acid profiles considered in this compilation

Fatty acid [%]	*Caulobacter* b *n* = 18			*Roseobacter* c *n* = 8			*Oceanicaulis* d *n* = 3			*Pelagibaca* e *n* = 3			*Pelagibacterales strain* HTCC7211
	Min	Max	% of *n* a	Min	Max	% of *n*	Min	Max	% of *n*	Min	Max	% of *n*	
10:0 3OH	—	—	—	1.8	4.2	100	—	—	—	0.0	13.3	67	—
12:1 *cis*5	—	—	—	—	—	—	—	—	—	—	—	—	4.1
12:0	0.0	4.9	72	—	—	—	—	—	—	0‐0	16.5	67	0.6
12:1 3OH	0.8	5.0	100	—	—	—	—	—	—	—	—	—	1.6
12:0 3OH	0.0	0.7	67	—	—	—	1.0	3.0	100	0.0	3.4	67	—
14:0	0.0	5.3	94	—	—	—	—	—	—	0.0	5.9	67	—
15:0	0.0	14.9	89	—	—	—	—	—	—	—	—	—	0.8
15:0 iso 3OH	—	—	—	—	—	—	—	—	—	—	—	—	0.5
16:1 *cis*9	0.4	20.3	100	0.0	1.0	50	0.0	0.9	67	—	—	—	45.8
16:0	11.7	29.9	100	1.1	2.2	100	0.8	4.9	100	4.9	16.5	100	17.7
16:0 iso 10methyl	—	—	—	—	—	—	—	—	—	—	—	—	0.5
17:0 cyclo9–10	—	—	—	—	—	—	—	—	—	—	—	—	11.9
17:1 *cis*9	0.0	5.3	67	—	—	—	0.0	2.0	67	—	—	—	—
17:1 *cis*11	0.0	8.9	61	—	—	—	0.0	6.1	67	—	—	—	—
17:0	0.0	14.4	61	—	—	—	6.9	25.1	100	—	—	—	—
18:0	—	—	—	0.0	2.8	50	11.1	29.3	100	—	—	—	1.3
18:1 *cis*11	19.3	57.6	100	84.6	93.0	100	27.8	41.4	100	32.4	79.7	100	—
18:1 *cis*11 11methyl	0.8	26.9	100	—	—	—	7.2	25.8	100	0.0	3.2	67	0.8
19:1 *cis*12	—	—	—	—	—	—	—	—	—	—	—	—	0.7
19:0 cyclo11–12	—	—	—	—	—	—	—	—	—	0.0	10.2	67	—
19:0	—	—	—	—	—	—	1.1	3.2	100	—	—	—	—

a Percentage of profiles containing this fatty acid; only fatty acids with presence in more than 40% of the profiles are listed.

b Data from (Abraham et al., 1999; Jin et al., 2014; 2013; Sun et al., 2015).

c Data from (Gosink, Herwig, & Staley, 1997; Labrenz et al., 1999; Martens et al., 2006).

d Data from (Chen, Sheu, Chen, Wang, & Chen, 2012; Zhang et al., 2013).

e Data from (Cho & Giovannoni, 2006; Lin et al., 2014).

### 3.4 *Synechococcus*

Depth profiles for the cyanobacterium *Synechococcus* were only taken in 2011. The relative abundance of this group increased with depth at all stations, however, the onset of the increase was found at a different depth between the stations. At the continental margin, the relative abundance of *Synechococcus* increased significantly from 2.3 ± 1.3% at 150 m to 5.0 ± 1.3% at 400 m (ANOVA; *p* < 0.01). A similar increase was observed at station CB from 2.7 ± 1.0% at 60 m to 6.0 ± 4.3% at 880 m (Figure 3b). Significantly higher numbers were found in the BL at 4,100 m with 8.7 ± 1.6% (ANOVA; *p* < 0.01; Figure 3b). A significant increase in the relative abundance of *Synechococcus* was found at station CBi from an average of 3.3% in the first 1,250 m of the water column to 5.6 ± 2.4% at 1,900 m and even 7.3 ± 1.0% at 2,600 m in the BL (ANOVA; *p* < 0.01; Figure 3b). The dominating fatty acids of unicellular cyanobacteria including *Synechococcus* are 16:0 and 16:1, even though *Synechococcus* clades 6–8 also contain high amounts of the polyunsaturated fatty acids 16:2 and 18:2 (Caudales, Wells, & Butterfield, 2000; Kenyon, 1972). The distribution of fatty acids 16:0 and 16:1 over the profiles was demonstrated in Figure 4 as sum of short chain fatty acids. These lipid markers are characteristic for many bacteria, not solely *Synechococcus*, and showed a decrease for most profiles with depths.

### 3.5 *Gammaproteobacteria*

Generally, slightly higher relative abundance of *Gammaproteobacteria* was found in 2011 as compared to 2010. The relative abundance of *Gammaproteobacteria* was, with the exception of station CB in 2011, highest in the surface waters and decreased immediately with depth (Figure 3b). At the continental margin, the relative abundance of *Gammaproteobacteria* was ~6% and decreased to ~2% below 45 m in both years (Figure 3a and b). On the contrary, the abundances of the subgroups *Alteromonas* and *Pseudoalteromonas* increased from <1% at the surface toward ~2% and ~2% with depth (Figure 3d). A similar decrease in the *Gammaproteobacteria* was found at station CBi, particularly during 2011, where surface waters showed the highest relative abundance of *Gammaproteobacteria*, 27.3 ± 1.8% at 53 m, decreasing to 3.0 ± 0.3% at 100 m depth (Figure 3b). In this year, two smaller increases were found within the INL (~7% between and 600 m) and the BL (11.4% between 2,150 m and 2,600 m) (Figure 3b). Similarly, *Alteromonas* and *Pseudoalteromonas* showed significantly higher abundance at 2,600 m depth with 14.0 ± 5.1% and 7.9 ± 3.8% (Figure 3b; *p* = 0.00162 and *p* = 0.0177). At station CB, *Gammaproteobacteria* showed a small peak of 6.4 ± 2.4% at 2,500 m, whereas in 2011, a relative abundance increased to 8.9 ± 4.6% at 1,000 m with a significant peak 18.2 ± 7.9% (ANOVA; *p* < 0.05) at 3,300 m (Figure 3a and b). Similarly, peaks of *Alteromonas* and *Pseudoalteromonas* with 7.8 ± 3.2% and 7.4 ± 2.6% where found at 880 m and 6.1 ± 0.6% and 6.2 ± 0.7% at 3,300 m (Figure 3b). The fatty acid data showed the decrease in the short straight chain fatty acids, characteristic for most *Gammaproteobacteria*, with depth for all profiles (Figure 4) and therefore confirmed the findings from the CARD‐FISH counts.

### 3.6 *Bacteroidetes*

As for most groups tested, the relative abundance of *Bacteroidetes* cells was slightly higher in 2011 than in 2010, with the exception of the continental margin, where the abundance at 20 m was significantly higher in 2010 (23.5 ± 2.2%) than in 2011 (14.3 ± 0.4%) and at depth (Figure 3a and b; ANOVA; 2010: *p* = 0.0387; 2011: *p* = 0.00372). At station CBi, a similar decrease from 15.3 ± 4.0% at 20 m and 17.6 ± 3.2% at 45 m toward an average of 4.2 ± 1.8% below 45 m was observed in 2010 (Figure 3a), whereas in 2011 the highest relative abundance (24.4 ± 0.1%) was found at 100 m depth (Figure 3b). At station CB, the abundance of *Bacteroidetes* did not change significantly with depth and was as low as 1.8%–5.8% in 2010 (Figure 3a and b). In contrast, the highest relative abundance was found at 400 m (16.6 ± 3.0%) and 880 m (18.1 ± 2.5%) in 2011 (Figure 3b). Two additional peaks of *Bacteroidetes* where found at 2,650 m at station CBi and at 3,300 m at station CB in 2011.

The fatty acid profiles of *Bacteroidetes* are dominated by iso/ante‐iso fatty acids, such as 15:0 iso, 15:0 ante‐iso, 16:0 iso, 17:0 iso, 17:0 ante‐iso fatty acid (Alain, Tindall, Catala, Intertaglia, & Lebaron, 2010; Kwon et al., 2018; Thongphrom, Kim, & Kim, 2016). This is in contrast to the straight chain fatty acids found in most *Alpha*‐ and *Gammaproteobacteria*. The abundances of these branched fatty acids were low throughout both transects (Figure 4) and resemble the low abundance of *Bacteroidetes* in the giant Cape Blanc filament.

### 3.7 Eukaryotic algae

The fatty acid profiles provide also some information about the distribution of eukaryotic algae. In contrast to bacteria, algae produce long chain fatty acids including polyunsaturated fatty acids (Wood, 1988). In Figure 4 the distribution of long chain fatty acids 18:0, 18:1 cis9, and 18:1 cis9,12 (eukaryotic fatty acids) demonstrates a general relative increase in these lipid markers with depths for all profiles.

## 4 DISCUSSION

In this study, we report the structure and distribution of the bacterial and archaeal communities along a transect in the CC off Cape Blanc (Mauretania) for two consecutive years using CARD‐FISH and FAME analyses. Here, the perennial phytoplankton bloom in the Cape Blanc filament of the CC shows highest intensities from January to June (Arístegui et al., 2009; Lathuilière et al., 2008). Turbidity and oxygen measurements showed that both, the NLs (INL, BL, and BL clouds) and the hypoxic waters were at the expected depths in both years (Fischer & Karakaş, 2009; Karakaş et al., 2006; Karstensen, Stramma, & Visbeck, 2008; Müller & Fischer, 2001), whereas the Chl a maximum was deeper and weaker in 2010 than in 2011, which might be caused by the earlier sampling time (February to March in 2010 vs. April to May in 2011) and a bloom that was not fully established in 2010. This might also influence bacterial abundances, which were higher in 2011 and might be due to higher bacterial activity and consequently better CARD‐FISH labeling. Low numbers indicate that the DAPI signals may not be exclusively of bacterial or archaeal origin, but may also include small eukaryotes, as previously noted for this region (Baltar et al., 2012), and may lead to an underestimation of coverage. In accord with this assumption we found a general decrease in short straight chain fatty acids for all profiles, which we associate with the decrease in bacteria. This corresponds well with the decrease in bacterial CARD‐FISH counts with depth for all profiles except for station CB in 2010. The opposite trend was found for eukaryotic fatty acids which increased with depths for all profiles, indicating an increase in eukaryotes as predicted by the decreasing coverage of bacterial and archaeal CARD‐FISH probes. It should also be noted that the fatty acid concentrations may be underestimated, since the filters for the lipid extractions had 0.7 μm pores. Hence, small cells could have been partially lost (Ingalls, Huguet, & Truxal, 2012). However, high particle loads in the system results in clogging of the filter during the filtration process, which will reduce the pore size and allow a mostly representative recovery of the microbial community. In the same samples intact polar membrane lipids of archaea, which usually have small cells, were detected, indicating that small cells were captured (Basse et al., 2014). The bacterial and archaeal depth profiles of the transect, including the negative correlation of bacterial and archaeal abundance at depth, are comparable to depth profiles from other oceanographic regions (Karner, DeLong, & Karl, 2001; Schattenhofer et al., 2009).

The most abundant heterotrophic bacteria in most marine systems are *Alphaproteobacteria*, mainly due to the high and ubiquitous abundance of the SAR11 clade which account for 20%–50% of all marine bacteria cells and occur at all depths throughout the global ocean (Brown et al., 2012; Morris et al., 2002; Schattenhofer et al., 2009; Thiele et al., 2012). In contrast to the finding of higher abundances for many bacterial groups in the NLs, the SAR11 clade was more often found at elevated abundances in the hypoxic waters, especially at the most oceanic site station CB, where SAR11 abundance and oxygen concentrations correlated negatively (*R*² = −0.0424 (2010) and *R*² = −0.0806 (2011)). Here, the highest relative abundance of SAR11 was found either on the upper (2010) or lower (2011) margin of the hypoxic zone. The presence of SAR11 in the CC hypoxic waters is congruent with previous findings of this group in OMZs (Lüke et al., 2016; Stevens & Ulloa, 2008; Tsementzi et al., 2016). Due to the high diversity within the SAR11 clade, varying subclades can be found at different depth or oceangraphic regions. In comparison to the surface dwelling and very abundant subclade Ia (Brown et al., 2012), the subclade IIa.A increases in abundance in OMZs (Tsementzi et al., 2016). The co‐occurance of the SAR11 clade and hypoxic waters could also explain the high abundances of SAR11 at the continental margin, where oxygen was low from ~100 m to the bottom.

While the SAR11 clade was found co‐occurring with hypoxic waters of the CC, *Roseobacter*,* Gammaproteobacteria*,* Bacteroidetes*, and *Synechococcus*, were found at the eutrophic surface with high Chl a values. *Roseobacter* are often found in correlation with phytoplankton blooms (Buchan, Gonzalez, & Moran, 2005; Rooney‐Varga et al., 2005), degrading the dissolved organic matter and metabolizing dimethylsulfoniopropionate (DMSP) released by many algae (Keller, Bellows, & Guillard, 1989), thus having an important role in the marine sulfur cycle. Similarly, *Gammaproteobacteria* groups, like *Alteromonas*,* Vibrio*, or *Pseudoalteromonas* prefer eutrophic waters (Sison‐Mangus, Jiang, Kudela, & Mehic, 2016; Teeling et al., 2016), which might explain the higher abundance in the surface and at the continental margin. Another major marine phylum often found to positively correlate with phytoplankton abundance and marine snow particles is *Bacteroidetes* (DeLong, Franks, & Alldredge, 1993; Glöckner, Fuchs, & Amann, 1999; Teeling et al., 2012; Thiele et al., 2015). Members of this highly diverse phylum have also been linked to the degradation of high molecular weight DOC (Cottrell & Kirchman, 2000; Gomez‐Pereira et al., 2010). Although for some profiles like station CB in 2011 high abundances of *Bacteroidetes* were found by CARD‐FISH counts, this could not be confirmed by fatty acid analyses. The characteristic branched chain fatty acids for this phylum were clearly below 10% for all samples analyzed.

*Synechococcus*, the second most abundant phototrophic bacteria in the ocean is usually found in the surface layer (Flombaum et al., 2013). However, in the CC it showed lower relative abundance in the surface water potentially due to competition with the bloom forming diatoms. On the contrary, *Synechococcus* was found to increase in relative abundance with depth, which seems contradictory to their phototrophic lifestyle. Still, evidence exists that *Synechococcus* might be able to survive heterotrophically in darkness (Cottrell & Kirchman, 2009; Zubkov, Fuchs, Tarran, Burkill, & Amann, 2003; Zubkov & Tarran, 2005). As previously shown for this stations, *Synechococcus* abundance increases with depth which is the result of attachment to sinking particles and subsequent transport into dark waters (Thiele et al., 2015). This attachment to particles would explain the high abundance in the BL clouds and the BL, where *Synechococcus* might also be transported horizontally from the continental shelf.

In addition, peaks of *Roseobacter*,* Gammaproteobacteria*, and *Bacteroidetes* were found at depths, where particles of the INL, BL clouds, or BL were abundant. Members of all these clades are known to attach to particles (Bižić‐Ionescu et al., 2015; DeLong et al., 1993). However, the abundance of *Alteromonas* and *Bacteroidetes* on particles was found to decrease from surface to 400 m depth indicating that the attached living community was inherited from surface waters (Thiele et al., 2015). This is congruent with the finding of *Bacteroidetes* at the depth of the NLs, since particles from the continental shelf could function as vectors for horizontal dispersal within the INL, the BL, and the BL clouds (Fischer et al., 2009; Karakaş et al., 2006), which is supported by high relative abundances of *Bacteroidetes* (~25%) at the continental shelf in 2010 (data not shown). In addition, particles in the NL might accumulate bacteria by attachment or cell growth over time and distance from the shore, which would also reflect the increase in relative abundance from the continental margin to station CB in the corresponding INL and BL depths. The increase in *Gammaproteobacteria* abundance at 3,300 m at Station CB in 2011 could hence indicate a small BL cloud that was not detectable using the turbidity sensor of the CTD, but was indicated by increased total organic carbon levels and the influx of North Atlantic Deep Water at this depth (Basse et al., 2014; Iversen et al., 2010). Attachment to particles and subsequent vertical transport within the biological pump or horizontal Ekman transport would provide a vehicle for bacterial dispersal over large distances. However, the correlation of particle transport, bacterial attachment, and consequently bacterial dispersal within the NLs requires further research.

## CONFLICT OF INTEREST

The authors declare no conflict of interest.

## DATA ACCESSIBILITY

CARD‐FISH and FAME data were submitted to the PANGAEA database and are available online (https://doi.org/10.1594/pangaea.889467).

## Supporting information


**  **

**  **

**  **
