# Supplementary material for: Microbial communities in the nepheloid layers and hypoxic zones of the Canary Current upwelling system
Source: Microbiologyopen. 2018 Oct 11;8(5):e00705. doi: 10.1002/mbo3.705 (PMC6528590; doi:10.1002/mbo3.705)
Supplement: Supplementary file 1 [file MBO3-8-e00705-s001.pdf]

Table S1: List of oligonucleotides used in this study. [a] Formamide concentration of the hybridization buffer, [b], used in a mix; [c] used in mix with BET42a (Manz et al. 1992) unlabeled as competitor.

| | **Target organism** | **Sequence (5'→ 3')** | ***E. coli* position** | **FA (%)[a]** | **Ref.** |
|---|---|---|---|---|---|
| **EUB 338[b]** | **Most *Bacteria*** | **GCTGCCTCCCGTAGGAGT** | **338-355** | **35** | **(Amann et al., 1990)** |
| **EUB II[b]** | ***Planctomycetales*** | **GCAGCCACCCGTAGGTGT** | **338-355** | **35** | **(Daims et al., 1999)** |
| **EUB III[b]** | ***Verrucomicrobiales*** | **GCTGCCACCCGTAGGTGT** | **338-355** | **35** | **(Daims et al., 1999)** |
| **NON338** | **Control** | **ACTCCTACGGGAGGCAGC** | **338-355** | **35** | **(Wallner et al., 1993)** |
| **ARCH915** | ***Archaea*** | **GTGCTCCCCCGCCAATTCCT** | **915-934** | **35** | **(Stahl & Amann 1991)** |
| **CREN554** | ***Thaumarchaeota*** | **TTAGGCCCAATAATCMTCCT** | **554-573** | **0** | **(Massana et al., 1997)** |
| **EURY806** | ***Euryarchaeota* marine group II** | **CACAGCGTTTACACCTAG** | **806-823** | **0** | **(Teira et al., 2004)** |
| **SAR11 441** | **SAR11 clade** | **AAAAAATACAGTCATTTCCTCCCCCGAC** | **441-463** | **25** | **(Rappe et al., 2002)** |
| **ROS537** | ***Roseobacter* clade** | **CAACGCTAACCCCCTCC** | **537-553** | **35** | **(Eilers et al., 2001)** |
| **GAM42a** | ***Gammaproteobacteria*** | **GCCTCCCCACATCGTTT** | **1027-1043** | **35** | **(Manz et al. 1992)** |
| **ALT1413** | ***Alteromonas / Colwellia*** | **TTTGCATCCCACTCCCAT** | **1413-1430** | **40** | **(Eilers et al. 2000b)** |
| **PSA184** | ***Pseudoalteromonas*** | **CCCCTTTGGTCCGTAGAC** | **184-201** | **30** | **(Eilers et al. 2000b)** |
| **CF319a** | ***Bacteroidetes*** | **TGGTCCGTGTCTCAGTAC** | **319-336** | **35** | **(Manz et al. 1996)** |
| **SYN405** | ***Synechococcus*** | **AGAGGCCTTCATCCCTCA** | **405-422** | **30** | **(West et al. 2001)** |

## References


Amann, R., Krumholz, L., Stahl, D., 1990. Fluorescent-oligonucleotide probing of whole cells for determinative, phylogenetic, and environmental studies in microbiology. J Bacteriol 172, 762–770.

Daims, H., Bruhl, A., Amann, R., Schleifer, K.H., Wagner, M., 1999. The domain-specific probe EUB338 is insufficient for the detection of all Bacteria: Development and evaluation of a more comprehensive probe set. Syst. Appl. Microbiol. 22, 434–444.

Eilers, H., Pernthaler, J., Peplies, J., Glöckner, F.O., Gerdts, G., Amann, R., 2001. Isolation of novel pelagic bacteria from the German Bight and their seasonal contributions to surface picoplankton. Appl Environ Microbiol 67, 5134–5142. https://doi.org/10.1128/AEM.67.11.5134-5142.2001

Eilers, H., Pernthaler, K., Glöckner, F.O., Amann, R., 2000. Culturability and in situ abundance of pelagic bacteria from the North Sea. Appl Environ Microbiol 66, 3044–3051. https://doi.org/10.1128/AEM.66.7.3044-3051.2000

Manz, W., Amann, R., Ludwig, W., Vancanneyt, M., Schleifer, K.H., 1996. Application of a suite of 16S rRNA-specific oligonucleotide probes designed to investigate bacteria of the phylum cytophaga-flavobacter-bacteroides in the natural environment. Microbiology 142, 1097–1106. https://doi.org/10.1099/13500872-142-5-1097

Manz, W., Amann, R., Ludwig, W., Wagner, M., Schleifer, K.H., 1992. Phylogenetic Oligodeoxynucleotide Probes for the Major Subclasses of Proteobacteria: Problems and Solutions. Syst Appl Microbiol 15, 593–600. https://doi.org/10.1016/S0723-2020(11)80121-9

Massana, R., Murray, A.E., Preston, C.M., DeLong, E.F., 1997. Vertical distribution and phylogenetic characterization of marine planktonic Archaea in the Santa Barbara Channel. Appl. Environ. Microbiol. 63, 50–56.

Rappe, M.S., Connon, S.A., Vergin, K.L., Giovannoni, S.J., 2002. Cultivation of the ubiquitous SAR11 marine bacterioplankton clade. Nature 418, 630–633. https://doi.org/10.1038/nature00917

Stahl, D.A., Amann, R., 1991. Development and application of nucleic acid probes, in: Stackebrandt, E., Goodfellow, M. (Eds.), Nucleic Acid Techniques in Bacterial Systematics. Chichester: John Wiley & Sons Ltd., pp. 205–248.

Teira, E., Reinthaler, T., Pernthaler, A., Pernthaler, J., Herndl, G.J., 2004. Combining catalyzed reporter deposition-fluorescence in situ hybridization and microautoradiography to detect substrate utilization by Bacteria and Archaea in the deep ocean. Appl Environ Microbiol 70, 4411–4414. https://doi.org/10.1128/AEM.70.7.4411-4414.2004

Wallner, G., Amann, R., Beisker, W., 1993. Optimizing fluorescent in situ hybridization with rRNA-targeted oligonucleotide probes for flow cytometric identification of microorganisms. Cytometry 14, 136–143.

West, N.J., Schönhuber, W.A., Fuller, N.J., Amann, R., Rippka, R., Post, A.F., Scanlan, D.J., 2001. Closely related Prochlorococcus genotypes show remarkably different depth distributions in two oceanic regions as revealed by in situ hybridization using 16S rRNA-targeted oligonucleotides. Microbiology 147, 1731–1744.
