# Supplementary material for: Microbial communities in the nepheloid layers and hypoxic zones of the Canary Current upwelling system
Source: Microbiologyopen. 2018 Oct 11;8(5):e00705. doi: 10.1002/mbo3.705 (PMC6528590; doi:10.1002/mbo3.705)
Supplement: Supplementary file 2 [file MBO3-8-e00705-s002.pdf]

Table S2A: CARD-FISH [% of DAPI counts] data of continental margin 2010

Taxonomic group (probe)	depth [m]							
	**20**	**45**	**70**	**100**	**200**	**300**	**500**	**700**
**Bacteria (EUB331)**	62.8	65.5	53.6		62.4	57.0	51.1	54.5
**Bacteroidetes (CF319a)**	23.6		4.0		2.6	2.8	1.8	4.9
**Roseobacter (ROS537)**	5.4	4.2	3.2	0.9	0.1	0.3	0.5	1.3
**SAR11 clade (SAR11 441)**	21.5	24.8	31.3		26.1	17.8	21.4	21.1
**Gammaproteobacteria (GAM42a)**	6.4	6.6	1.8		0.6	2.3	1.9	2.0
**Alteromonas/ Colwellia (ALT1413)**								
**Pseudoalteromonas (PSA184)**								
**Synechrococcus (SYN405)**								
**Thaumarchaeota (CREN554)**		5.7	20.6	28.0	26.4	30.4	18.3	28.4
**Euryarchaeota (EURY806)**		5.3	2.8	6.5	3.5	3.6	1.3	2.3

Table S2B: CARD-FISH [% of DAPI counts] data of Station CBi 2010

Taxonomic group (probe)	depth [m]								
	**20**	**45**	**150**	**300**	**600**	**1000**	**1900**	**2200**	**2650**
**Bacteria (EUB331)**	53.4	63.2	41.7	58.6	44.6	41.0	36.4	45.0	46.0
**Bacteroidetes (CF319a)**	15.3	17.7	5.0	3.1	1.8	3.5	6.3	6.5	2.9
**Roseobacter (ROS537)**	3.9	4.0	2.9	1.2	1.6	0.4	0.3	0.6	0.4
**SAR11 clade (SAR11 441)**	35.8	45.1	17.2	18.2	17.7	15.1	10.7	12.1	4.3
**Gammaproteobacteria (GAM42a)**	4.7	3.1	2.7	3.2	3.3	3.0	3.4	3.2	2.7
**Alteromonas/ Colwellia (ALT1413)**									
**Pseudoalteromonas (PSA184)**									
**Synechrococcus (SYN405)**									
**Thaumarchaeota (CREN554)**	0.7	7.4	26.4	28.9	20.5	18.5	24.4	14.4	18.0
**Euryarchaeota (EURY806)**	6.1	8.9	2.3	2.5	1.4	0.8	1.5	1.6	0.9

Table S2C: CARD-FISH [% of DAPI counts] data of Station CB 2010

Taxonomic group (probe)	depth [m]										
	**20**	**45**	**100**	**200**	**400**	**500**	**700**	**1500**	**2500**	**3500**	**4100**
**Bacteria (EUB331)**	58.5	63.4	46.1	43.4	48.1	50.5	36.8	55.7	65.2	55.6	64.0
**Bacteroidetes (CF319a)**	5.8	6.0	3.4	3.1	2.0	2.7	1.8	3.4	5.7	3.8	1.8

**Roseobacter (ROS537)**	1.2	0.3	0.5	1.5	0.3	0.4	1.9	2.2	1.1	0.4	0.5
**SAR11 clade (SAR11 441)**	7.5	5.1	4.4	5.8	12.0	6.2	4.6	6.5	4.1	4.1	5.4
**Gammaproteobacteria (GAM42a)**	7.1	3.8	2.1	2.7	2.3	1.9	2.1	4.1	6.4	3.1	4.1
**Alteromonas/ Colwellia (ALT1413)**											
**Pseudoalteromonas (PSA184)**											
**Synechrococcus (SYN405)**											
**Thaumarchaeota (CREN554)**	1.6	0.6	4.5	2.1	2.1	3.7	2.8	3.2	4.6	7.5	3.6
**Euryarchaeota (EURY806)**	2.4	1.1	1.6	0.9	1.4	1.0	0.4	2.1	0.9	0.6	0.3

Table S2D: CARD-FISH [% of DAPI counts] data of continental margin 2011

**Taxonomic group (probe)**	**depth [m]**				
	**20**	**150**	**350**	**450**	**550**
**Bacteria (EUB331)**	86.2	78.8	70.4	72.8	70.0
**Bacteroidetes (CF319a)**	14.2	3.7	2.8	3.2	3.4
**Roseobacter (ROS537)**	3.4	1.5	1.0	1.6	1.4
**SAR11 clade (SAR11 441)**	23.4	14.5	16.6	13.2	12.5
**Gammaproteobacteria (GAM42a)**	6.3	2.9	3.3	3.5	4.0
**Alteromonas/ Colwellia (ALT1413)**	0.9	1.6	2.2	2.9	1.2
**Pseudoalteromonas (PSA184)**	0.9	2.6	1.9	2.3	2.3
**Synechrococcus (SYN405)**	1.4	2.3	5.0	4.8	4.3
**Thaumarchaeota (CREN554)**	1.5	20.9	21.4	21.5	21.9
**Euryarchaeota (EURY806)**					

Table S2E: CARD-FISH [% of DAPI counts] data of Station CBi 2011

**Taxonomic group (probe)**	**depth [m]**									
	**53**	**100**	**250**	**350**	**600**	**910**	**1250**	**1900**	**2150**	**2600**
**Bacteria (EUB331)**	60.2	44.3	32.1	37.4	36.4	34.6	33.5	33.0	36.1	32.5
**Bacteroidetes (CF319a)**		24.4	14.6	8.5	5.0	9.9	9.0	8.6	8.1	14.0
**Roseobacter (ROS537)**	3.2	1.8	1.9	1.5	1.6	2.5	2.6	2.9	3.4	6.8
**SAR11 clade (SAR11 441)**	17.0	12.5	16.3	13.1	22.6	17.4	15.8	9.8	11.3	9.8
**Gammaproteobacteria (GAM42a)**	27.3	3.1	6.5	7.1	8.6	4.2	3.5	4.3	11.2	11.6

**Alteromonas/ Colwellia (ALT1413)**	2.5	2.3	2.4	1.8	1.7	3.7	2.2	3.6	3.5	14.0
**Pseudoalteromonas (PSA184)**	3.9	3.1	2.5	3.1	2.6	3.1	1.8	5.1	3.2	7.9
**Synechrococcus (SYN405)**	3.1	2.5	3.5	2.8	3.7	3.7	3.6	5.7	4.7	7.3
**Thaumarchaeota (CREN554)**	8.1	8.8	25.2	23.0	22.4	25.7	20.1	12.3	12.7	14.9
**Euryarchaeota (EURY806)**	60.2	44.3	32.1	37.4	36.4	34.6	33.5	33.0	36.1	32.5

Table S2F: CARD-FISH [% of DAPI counts] data of Station CB 2011

**Taxonomic group (probe)**	**depth [m]**									
	**60**	**130**	**250**	**400**	**880**	**1000**	**1500**	**2150**	**3300**	**4100**
**Bacteria (EUB331)**	56.2	32.9	38.6	41.2	49.6	44.7	38.1	34.9	41.4	28.4
**Bacteroidetes (CF319a)**		9.0	8.7	16.6	18.1	12.3	11.2	6.9	15.8	9.8
**Roseobacter (ROS537)**	2.3	1.8	3.5	1.6	4.2	5.6	5.2	3.3		6.2
**SAR11 clade (SAR11 441)**	14.9	13.7	13.2	10.6	13.3	19.5	9.1	9.5	10.0	9.9
**Gammaproteobacteria (GAM42a)**	2.5	2.6	3.9	3.5	4.4	8.9	5.4	5.3	18.2	5.9
**Alteromonas/ Colwellia (ALT1413)**	0.9	2.8	3.8	1.5	7.8	4.9	3.0	3.9	6.1	3.2
**Pseudoalteromonas (PSA184)**	1.2	2.4	2.7	1.9	7.4	3.4	5.3	3.2	6.2	3.9
**Synechrococcus (SYN405)**	2.7	2.6	2.7	3.7	6.0	6.2	5.0	6.0	6.4	8.7
**Thaumarchaeota (CREN554)**	4.9	14.7	18.1	20.4	9.4	19.9	9.2	18.5	11.0	8.9
**Euryarchaeota (EURY806)**	56.2	32.9	38.6	41.2	49.6	44.7	38.1	34.9	41.4	28.4
