# Supplementary material for: Microbial communities in the nepheloid layers and hypoxic zones of the Canary Current upwelling system
Source: Microbiologyopen. 2018 Oct 11;8(5):e00705. doi: 10.1002/mbo3.705 (PMC6528590; doi:10.1002/mbo3.705)
Supplement: Supplementary file 3 [file MBO3-8-e00705-s003.pdf]

Table S3A: Fatty acid profiles of continental margin 2010

Fatty acid [%]	depth [m]					
	45	100	170	300	600	720
**14:0**	13.1	3.9	1.0	3.9	0.2	0.1
**15:0 iso**	1.4	1.6	0.7	1.6	0.1	0.1
**15:0 anteiso**	0.6	0.8	0.4	1.0	0.1	0.0
**15:0**	1.5	2.4	1.0	2.3	0.0	0.0
**16:0 10-methyl**	0.6	0.4	0.2	0.4	0.2	0.0
**16:0 iso**	0.7	0.9	0.6	0.8	0.4	0.1
**16:1 cis9**	13.8	8.3	3.2	10.9	3.7	0.9
**16:1 cis11**	0.9	0.8	0.5	1.2	0.4	0.1
**16:0**	36.9	41.4	30.9	36.1	25.2	8.2
**17:0 cyclo**	0.5	2.5	3.4	3.4	3.4	2.2
**17:0**	0.9	2.1	2.7	1.6	1.8	1.3
**18:2 cis9,12**	2.6	2.8	2.8	2.2	4.2	2.9
**18:1 cis9**	7.5	8.5	7.6	13.4	25.6	27.2
**18:1 cis11**	7.5	5.3	6.4	6.5	6.7	10.9
**18:0**	4.9	8.6	17.5	6.5	11.3	15.4
**18:2**	0.3	0.6	0.9	0.6	1.2	1.5
**19:0 cyclo**	0.1	0.7	2.4	1.0	1.2	1.8
**19:0**	0.2	0.5	1.4	0.3	0.6	0.9
**20:2**	2.5	1.1	0.2	1.2	1.6	2.1
**20:1**	0.4	0.6	1.1	0.4	3.3	6.3
**20:0**	1.6	2.0	3.9	0.9	1.7	3.1
**21:0**	0.1	0.2	0.5	0.4	0.4	0.8
**22:6**	0.0	0.0	0.0	0.0	0.0	0.1
**22:0**	0.6	1.4	4.8	1.2	3.1	5.2
**23:0**	0.1	0.3	0.9	0.2	0.5	0.8
**24:0**	0.5	1.4	2.6	1.0	1.7	3.9
**25:0**	0.0	0.2	0.7	0.2	0.4	0.8
**26:0**	0.1	0.4	0.9	0.4	0.7	1.8
**27:0**	0.1	0.2	0.6	0.3	0.4	0.9
**28:0**	0.0	0.1	0.2	0.1	0.2	0.5
**Total amount [ng/L]**	62160	15190	3179	9457	2638	1680

Table S3B: Fatty acid profiles of Station CBi 2010

Fatty acid [%]	depth [m]							
	45	150	300	1000	1900	2000	2200	2620
**14:0**	15.1	4.2	1.8	0.4	0.0	0.0	0.0	0.0
**15:0 iso**	0.7	1.9	1.3	0.5	0.0	0.0	0.0	0.0
**15:0 anteiso**	0.7	1.0	0.9	0.5	0.0	0.1	0.0	0.2
**15:0**	1.5	2.5	2.0	1.1	0.0	0.0	0.1	0.6
**16:0 10-methyl**	0.2	0.4	0.5	0.2	0.1	0.0	0.0	0.2
**16:0 iso**	0.3	0.9	0.8	0.7	0.3	0.0	0.1	0.6
**16:1 cis9**	9.9	6.3	7.2	2.9	1.0	0.2	0.3	2.7
**16:1 cis11**	0.5	0.6	0.8	0.3	0.0	0.0	0.0	0.3
**16:0**	46.6	44.2	41.7	28.9	8.8	0.7	4.8	26.6
**17:0 cyclo**	0.9	3.3	5.0	3.4	2.0	0.4	0.9	2.3

**17:0**	0.9	1.9	1.9	2.1	1.9	0.3	1.1	2.7
**18:2 cis9,12**	3.0	2.1	1.8	1.5	0.9	0.4	1.1	1.7
**18:1 cis9**	6.3	6.3	7.6	7.9	7.7	1.8	9.6	9.3
**18:1 cis11**	3.3	4.9	6.1	0.4	8.4	1.7	0.4	0.5
**18:0**	5.0	8.8	8.2	15.4	22.6	8.0	12.8	22.0
**18:2**	0.3	0.0	0.4	0.3	0.8	0.1	0.6	0.2
**19:0 cyclo**	0.3	1.0	1.5	1.3	1.7	0.8	0.9	0.8
**19:0**	0.2	0.5	0.5	0.7	1.6	0.5	0.7	1.1
**20:2**	0.6	0.7	0.9	0.4	0.0	0.1	0.2	0.1
**20:1**	0.1	0.4	0.6	0.9	1.5	0.2	2.4	0.8
**20:0**	1.3	1.8	1.3	2.2	4.3	2.0	2.3	3.4
**21:0**	0.2	0.3	0.2	0.5	1.0	0.5	0.5	0.7
**22:6**	0.0	0.1	0.0	0.0	0.3	0.4	0.5	0.2
**22:0**	1.0	2.4	4.5	16.9	17.6	74.5	50.8	7.1
**23:0**	0.1	0.3	0.2	1.0	1.6	0.7	0.7	1.4
**24:0**	0.7	1.6	1.3	4.5	8.4	3.2	4.5	7.1
**25:0**	0.1	0.3	0.2	1.1	1.8	0.9	0.9	1.7
**26:0**	0.2	0.8	0.5	2.4	3.8	1.6	2.3	3.7
**27:0**	0.1	0.3	0.2	0.5	0.6	0.3	0.4	0.7
**28:0**	0.0	0.2	0.2	1.1	1.3	0.4	0.8	1.2
**Total amount [ng/L]**	65984	11295	4111	1393	603	1463	1234	902

Table S3C: Fatty acid profiles of Station CB 2010

**Fatty acid [%]**	**depth [m]**						
	**45**	**200**	**700**	**1500**	**2500**	**3500**	**4120**
**14:0**	2.4	1.4	0.9	0.0	0.0	0.0	0.0
**15:0 iso**	0.3	1.0	0.5	0.1	0.0	0.0	0.2
**15:0 anteiso**	0.3	0.8	0.4	0.1	0.1	0.0	0.4
**15:0**	1.0	1.8	1.0	0.3	0.1	0.1	0.8
**16:0 10-methyl**	0.2	0.4	0.3	0.0	0.1	0.0	0.2
**16:0 iso**	0.1	0.7	0.7	0.7	0.2	0.2	0.6
**16:1 cis9**	7.1	3.8	4.0	2.9	0.4	1.0	2.9
**16:1 cis11**	0.6	0.5	0.4	0.3	0.1	0.1	0.2
**16:0**	50.5	30.1	29.0	15.8	10.2	13.7	20.8
**17:0 cyclo**	1.0	4.0	3.9	2.2	1.7	1.2	0.8
**17:0**	1.4	2.0	1.9	1.9	2.0	1.4	2.2
**18:2 cis9,12**	2.1	1.6	2.1	1.8	2.0	1.7	1.5
**18:1 cis9**	8.2	8.7	23.9	12.9	14.6	42.2	9.4
**18:1 cis11**	6.4	4.9	0.3	5.0	6.2	0.6	4.0
**18:0**	9.8	11.2	18.1	29.5	38.7	19.0	19.6
**18:2**	0.7	0.8	0.7	0.6	0.1	2.1	0.2
**19:0 cyclo**	0.5	3.9	1.2	1.4	0.8	0.9	0.2
**19:0**	0.5	0.5	0.6	1.3	1.3	0.6	0.8
**20:2**	0.3	0.2	1.2	0.3	0.2	0.4	0.0
**20:1**	0.3	0.4	1.7	3.8	3.5	6.1	0.6
**20:0**	2.1	1.2	1.6	3.8	3.6	1.8	2.7
**21:0**	0.8	0.3	0.3	0.9	1.0	0.3	0.5
**22:6**	0.1	2.6	0.1	0.1	0.1	0.2	0.2

**22:0**	1.9	4.4	2.6	7.4	8.2	3.5	23.6
**23:0**	0.2	2.9	0.4	1.8	1.1	0.3	0.7
**24:0**	0.9	0.8	1.2	2.9	2.6	1.5	4.1
**25:0**	0.1	1.1	0.2	1.1	0.5	0.3	0.9
**26:0**	0.2	2.9	0.3	0.7	0.6	0.4	1.4
**27:0**	0.2	2.7	0.3	0.0	0.0	0.2	0.2
**28:0**	0.1	2.5	0.1	0.0	0.0	0.0	0.2
**Total amount [ng/L]**	18207	5527	1217	378	571	738	1186

Table S3D: Fatty acid profiles of continental margin 2011

**Fatty acid [%]**	**depth [m]**			
	**150**	**350**	**450**	**550**
**14:0**	2.4	1.3	0.5	0.3
**15:0 iso**	1.7	0.9	0.6	0.2
**15:0 anteiso**	0.7	0.5	0.3	0.1
**15:0**	2.0	1.5	1.0	0.6
**16:0 10-methyl**	1.1	0.8	0.7	0.3
**16:1 cis9**	16.6	12.3	8.5	11.4
**16:1 cis11**	0.4	0.9	0.8	1.6
**16:0**	44.1	44.8	46.7	43.2
**17:0 iso**	1.3	0.4	0.5	0.4
**17:0 anteiso**	0.4	0.6	0.5	0.4
**17:0 cyclo**	2.8	2.8	2.5	0.7
**17:0**	2.0	1.6	1.5	1.5
**18:1 cis9**	5.5	11.2	12.5	10.5
**18:1 cis11**	8.3	5.3	5.5	4.2
**18:0**	6.5	7.8	9.1	12.9
**19:0 cyclo**	0.0	0.9	1.2	0.4
**19:0**	0.8	0.8	0.7	0.2
**20:2**	0.0	1.4	1.2	2.3
**20:1**	0.0	0.3	0.3	0.5
**20:0**	0.0	1.1	1.4	2.8
**21:0**	0.0	0.3	0.3	0.3
**22:6**	0.0	0.6	0.6	0.5
**22:1**	0.0	0.3	1.1	1.2
**22:0**	1.1	0.6	0.6	1.0
**23:0**	0.0	0.2	0.2	0.2
**24:0**	2.0	0.8	0.9	1.4
**25:0**	0.2	0.0	0.0	0.0
**26:0**	0.0	0.3	0.4	0.7
**Total amount [ng/L]**	138	193	105	356

Table S3E: Fatty acid profiles of Station CB1 2011

**Fatty acid [%]**	**depth [m]**				
	**53**	**350**	**1250**	**1900**	**2150**
**14:0**	7.4	2.1	1.1	2.4	0.0
**15:0 iso**	0.5	1.5	1.1	0.1	0.0

**15:0 anteiso**	0.4	0.9	0.8	1.5	0.0
**15:0**	1.3	2.0	1.7	2.6	0.0
**16:0 10-methyl**	0.2	0.9	1.2	1.3	0.0
**16:1 cis9**	4.8	7.7	10.3	6.0	1.6
**16:1 cis11**	1.9	4.3	1.5	3.1	8.0
**16:0**	52.9	42.5	33.5	39.8	10.0
**17:0 iso**	0.3	0.5	1.3	2.5	3.1
**17:0 anteiso**	0.8	1.6	0.7	0.0	5.6
**17:0 cyclo**	0.8	2.9	3.9	4.1	2.8
**17:0**	1.4	1.9	1.7	1.7	1.4
**18:1 cis9**	4.2	8.4	9.5	3.8	1.8
**18:1 cis11**	2.1	4.4	7.2	3.0	0.0
**18:0**	9.3	9.4	10.3	17.1	32.8
**19:0 cyclo**	0.5	0.7	1.1	2.0	4.9
**19:0**	0.3	0.9	1.2	0.7	0.0
**20:2**	0.0	0.9	2.6	0.0	0.0
**20:1**	4.2	0.0	0.6	0.0	0.0
**20:0**	0.7	2.8	1.4	2.3	9.9
**21:0**	0.6	0.4	0.7	0.0	3.2
**22:6**	0.4	0.0	1.1	1.9	5.7
**22:1**	0.0	0.8	1.9	0.6	0.0
**22:0**	1.9	0.9	0.9	1.0	2.5
**23:0**	0.4	0.0	0.5	0.0	0.0
**24:0**	2.0	1.6	1.4	2.5	6.7
**25:0**	0.0	0.0	0.1	0.0	0.0
**26:0**	0.7	0.0	0.8	0.0	0.0
**Total amount [ng/L]**	1246	85	32	23	19

Table S3F: Fatty acid profiles of Station CB 2011

**Fatty acid [%]**	**depth [m]**					
	**60**	**130**	**390**	**880**	**2150**	**3300**
**14:0**	1.2	2.1	1.4	0.7	0.0	0.1
**15:0 iso**	0.9	1.1	0.8	0.6	0.0	0.1
**15:0 anteiso**	2.4	3.2	2.1	1.9	0.0	0.4
**15:0**	0.3	1.2	0.9	1.0	0.0	0.2
**16:0 10-methyl**	15.9	11.4	13.5	11.5	8.0	5.2
**16:1 cis9**	0.2	1.5	0.9	1.1	1.9	1.0
**16:1 cis11**	59.8	46.5	38.9	36.2	17.3	17.0
**16:0**	0.2	0.5	0.6	1.0	2.9	1.7
**17:0 iso**	0.1	0.6	0.8	0.9	5.4	3.9
**17:0 anteiso**	1.1	3.4	3.7	4.2	3.1	1.9
**17:0 cyclo**	1.2	2.2	1.9	1.8	2.1	1.4
**17:0**	4.3	7.0	9.4	9.3	0.8	0.5
**18:1 cis9**	2.9	6.4	7.2	5.3	0.3	0.2
**18:1 cis11**	5.2	6.3	8.3	9.9	22.4	36.4
**18:0**	0.0	0.7	0.8	1.2	3.5	2.4
**19:0 cyclo**	0.4	1.2	1.1	0.9	2.0	1.1
**19:0**	0.0	0.5	1.5	2.3	0.0	0.0

**20:2**	0.0	0.0	0.3	0.8	0.0	0.0
**20:1**	1.9	1.3	1.6	3.7	10.6	7.9
**20:0**	0.2	0.0	0.0	0.0	3.1	3.1
**21:0**	0.2	1.6	1.8	0.9	0.0	2.8
**22:6**	0.0	0.0	0.5	2.2	1.9	0.0
**22:1**	0.7	0.6	0.7	0.8	4.7	2.0
**22:0**	0.1	0.0	0.2	0.2	2.2	1.9
**23:0**	0.6	0.7	0.8	1.0	4.2	4.8
**24:0**	0.0	0.0	0.0	0.1	0.8	0.8
**25:0**	0.2	0.0	0.3	0.4	2.9	3.3
**26:0**	1.2	2.1	1.4	0.7	0.0	0.1
**Total amount [ng/L]**	818	138	96	39	29	19
